# NUDGING ATTITUDE CHANGE THROUGH EXPERIENCE: UNDERGRADUATE CURRICULAR INNOVATIONS

**DOI:** 10.1093/geroni/igad104.0367

**Published:** 2023-12-21

**Authors:** Brian Carpenter

## Abstract

Drawing students into gerontology and geriatrics remains a perennial challenge in light of negative stereotypes and ageism deeply entrenched in society. Nonetheless, instructors continue to forge ahead with curricular innovations designed to dismantle stereotypes, promote deeper understanding of the later stage of life, cultivate appreciation for the contributions of older adults, and entice students to consider further academic, extracurricular, and vocational opportunities in the field. This symposium brings together scholars and instructors who have implemented and evaluated course innovations with these goals in mind. The first presentation reviews findings from the Bringing Art to Life program that pairs teams of undergraduate students with persons with dementia attending an adult day services program and engaging in structured art therapy and reminiscence. The next presenter describes positive attitude change about aging among undergraduates in an advanced public health service-learning course. Next is a discussion of how health policy education has been successfully built into an undergraduate adult development and aging course. The fourth presentation summarizes results from a randomized educational intervention to undergraduate students to enhance death education and ensure more equitable access to quality end-of-life services and supports. The final presentation describes innovations in training MSTEM undergraduate students in aging. A Discussant will synthesize themes across presentations and engage session attendees to respond and share their own curricular experiences.

